# Lobaplatin promotes ^125^I-induced apoptosis and inhibition of proliferation in hepatocellular carcinoma by upregulating PERK-eIF2α-ATF4-CHOP pathway

**DOI:** 10.1038/s41419-019-1918-1

**Published:** 2019-10-03

**Authors:** Dong Li, Wu-jie Wang, Yong-zheng Wang, Yi-biao Wang, Yu-liang Li

**Affiliations:** 1 Department of Interventional Medicine, The Second Hospital of Shandong University, Jinan, China; 2 Department of Pediatrics, The Second Hospital of Shandong University, Jinan, China

**Keywords:** Chemotherapy, Radiotherapy, Liver cancer

## Abstract

We investigated the mechanism underlying the effect of a combination treatment of ^125^I radioactive seed implantation and lobaplatin (LBP) in hepatocellular carcinoma. The effects of administration of HCC cells and subcutaneous tumor model of mice with different doses of ^125^I or a sensitizing concentration of LBP alone, or in combination, on cellular apoptosis and proliferation were analyzed and it was confirmed that LBP promotes ^125^I-induced apoptosis and inhibition of proliferation of HCC. Furthermore, isobaric tag for relative and absolute quantification labeling analyses suggested that ^125^I promoted the apoptosis and inhibition of proliferation of HCC cells by upregulating the expression of PERK-eIF2α-ATF4-CHOP pathway, a well-known apoptosis-related pathway. Moreover, LBP was found to boost the ^125^I-induced upregulation of this pathway and increase the apoptosis. Our data indicate that LBP promotes the apoptotic and anti-proliferative effects of ^125^I and provide a firm foundation for better clinical application of this combination therapy.

## Introduction

Hepatocellular carcinoma (HCC) is the third most common malignant cancer in China and has a serious negative effect on patient’s health. More than three million people die from HCC every year in China, especially in rural areas^1^. For inoperable HCC patients, radiotherapy (RT) alone does not improve the overall survival. Recently, ^125^I seed implantation has been proven to be a safe, efficacious, and economical method for treating moderate and advanced HCC. However, RT when combined with other treatments, such as platinum chemotherapy, exhibits a better prognosis than the non-RT therapies^2,3^. Due to the mechanisms underlying the effects of ^125^I seed in HCC and enhancement of the radiosensitivity of HCC to ^125^I seed by chemotherapy are unclear, identification of new cellular targets of ^125^I seed would lay a solid foundation for better clinical application of ^125^I seed implantation therapy and would provide novel therapeutic approaches for treating HCC.

Endoplasmic reticulum (ER) is an important organelle in cells. Damage of its function causes stress reaction in ER, which is known as ER stress. ER protects cells from the damage caused by such stress, by activating the unfolded protein response (UPR)^4,5^. The UPR relies on the duration of exposure of cells to unfavorable conditions, such as radiation, which may have disparate outcomes, such as adaptation to the stress or apoptosis^6^. A proper UPR aims to reduce the ER capacity and protein synthesis, causing the cells to adapt to the stress. However, in the event of an insufficient adaptive response, ER stress induces cells to go through apoptosis and regulates C/EBP homologous protein (CHOP), JNK activation, and Bcl-2 expression^7^. The PERK-eIF2α-ATF4-CHOP pathway plays an important role in ER stress; it induces apoptosis through upregulation of CHOP, Bcl-2, and other apoptosis-related factors.

As a third-generation platinum drug, lobaplatin (LBP) is reported to induce apoptosis and cell cycle arrest, and impairs the migration and invasion in various gastrointestinal tumor cell lines in vitro^8,9^. Cells at the G2/M transition stage are more sensitive to RT, indicating that LBP might enhance the radiosensitivity of HCC and ultimately decrease the biologically effective dose, serving to reduce RT-related complications^10,11^. A retrospective study showed that transarterial chemoembolization (TACE) with gelatin sponge microparticles mixed with LBP is a safe and effective method for stage B HCC patients^12^. Moreover, Peng et al.^13^ reported that the combination of LBP-TACE and brachytherapy has a better overall survival than that of LBP-TACE alone; thus, a comprehensive therapy is recommended for these patients^13^.

Based on the results of isobaric tag for relative and absolute quantification labeling (iTRAQ) and the function of PERK-eIF2α-ATF4-CHOP pathway, we hypothesized that ^125^I seeds might induce the upregulation of PERK-eIF2a-ATF4-CHOP pathway, resulting in apoptosis in liver cancer cells. Moreover, we verified that LBP could enhance the apoptosis and anti-proliferative activity of ^125^I, and assumed that this enhancement might work by regulating the PERK-eIF2α-ATF4-CHOP pathway. To test these hypotheses, the correlation between ^125^I and PERK-eIF2α-ATF4-CHOP pathway was evaluated in liver cancer cell lines and mice tumor model. We found that the PERK-eIF2α-ATF4-CHOP pathway was inhibited in liver cancer cells after treatment with ^125^I and LBP. Our results indicate that ^125^I induces the upregulation of PERK-eIF2a-ATF4-CHOP pathway to promote apoptosis and LBP promotes ^125^I-induced apoptosis by increasing the ^125^I-induced upregulation of PERK-eIF2α-ATF4-CHOP pathway. In summary, our data identify PERK-eIF2a-ATF4-CHOP pathway as a new mechanism of apoptosis induced by ^125^I and suggest that PERK-eIF2a-ATF4-CHOP pathway could be a new therapeutic target in ^125^I seed implantation therapy for HCC.

## Materials and methods

### Mice subcutaneous tumor formation assay

For xenograft tumor studies, 100 μl of SMMC7721 cells (1 × 10^7^/ml) transfected with PERK-RNAi or Control-RNAi were diluted in 0.9% saline solution and injected subcutaneously in the hind leg of BALB/c male mice (purchased from the Animal Research Center of Shandong University). When the volume of tumor reached 500 mm^3^, the mice were randomly divided into three group with four mice in each group. Tumor diameters and weight were measured every other day for 30 days, at which time mice were killed and tumors were excised, measured, and lysed for RNA isolation. All mice were housed under specific pathogen-free conditions and were killed according to regulations formulated by the Shandong University Animal Care Committee.

### Procedure for radioactive ^125^I seeds implantation in mice

First of all, the skin on the surface of the tumor was sterilized with iodine. After local anesthesia with lidocaine, the subcutaneous tumor was punctured with an 18G needle (Kakko, Japan) under direct vision. The endpoint of the needle was located at the middle of the tumor. The seed was advanced into the tumor through the needle using seed implantation equipment (Ningbo Junan Pharmaceutical Technology Company, Nanjing, China). When the procedure was completed, a sterile gauze was pressed to stop the bleeds, if necessary.

### Radiation for HCC cell lines

^125^I seeds were purchased from Ningbo Junan Pharmaceutical Technology Company (Ningbo, Zhejiang, China). ^125^I seeds were seeded into an in vitro irradiation model, based on a previous method^14^. The initial activity and dose rate were 3.0 mCi and 3.412 cGy/h, respectively. To deliver doses of 1, 2, and 4 Gy, irradiation times of 29.3, 58.6, and 117.2 h, respectively, were used.

### Cell lines and transfection

The HCC cell lines were purchased from the Chinese Academy of Sciences (Shanghai, China). The cells were incubated in 5% CO_2_ at 37 °C and grown in culture media supplemented with 10% fetal calf serum (Gibco, Waltham, MA USA). Human HCC cell line HepG2 was cultured in Dulbecco’s modified Eagle’s medium (DMEM; Corning, Inc., Corning, NY, USA). Human HCC cell line SMMC7721 was cultured in RPMI 1640 (Corning, Inc., Corning, NY, USA).

Transfections were performed using Lipofectamine 2000 (Invitrogen, CA, USA) according to the manufacturer’s instructions. A short interfering RNA (siRNA) for PERK (protein kinase RNA-like ER kinase) was designed by RiboBio (Guangzhou, Guangdong, China). PERK-RNAi was purchased from Genechem (Shanghai, China).

### iTRAQ labeling

HepG2 cells were treated with or without ^125^I and total protein was isolated with RIPA (TIANGEN). The iTRAQ was performed by Jiyun Biotech (Shanghai, China) in ^125^I-treated HepG2 cells and control HepG2 cells. The signal pathway enrichment was analyzed using Ingenuity Pathway Analysis (INGENUITY). The mean value between the ^125^I-treated group and control group, which is >1.2 and *p* < 0.05, is supposed to be significantly upregulated.

### Selection for sensitizing concentration of LBP to HCC cells

HCC cells were seeded in a 96-well plate. After treatment with concentrations of LBP ranging from 0 to 80 µg/mL for 72 h, the optical density was detected using CCK-8 assays (Dojindo, Kumamoto, Japan). The half-maximal inhibitory concentration (IC_50_) was calculated with Prism 6.0 (GraphPad, San Diego, CA, USA). According to the toxic effects of LBP on HCC cells and previously published method, the LBP-sensitizing concentration was determined to be 10% of the IC_50_^15^.

### Cell proliferation assay

After treatment, cells were collected as a single cell suspension and were seeded in 96-well plates at 5 × 10^3^ cells/well, with each well containing 200 µL culture medium. The CCK-8 reagent was added into the medium at 24, 48, and 72 h after the cell was attached and then incubated at 37 °C for 3 h. Finally, the plate was scanned by a microplate reader (Thermo, Waltham, MA, USA).

### Cell cycle and apoptosis analysis

Cells were seeded into six-well plates at 2 × 10^5^ cells per well and were treated as indicated. For the cell cycle assay, after collection, the cells were washed with cold phosphate-buffered saline (PBS), fixed with 70% cold ethanol, and stored at −4 °C overnight. Before analysis, the cells were stained with propidium iodide (PI) and RNase A (BD Biosciences, Franklin Lakes, NJ, USA). For the apoptosis analysis, after collecting, cells were either stained with PI and Annexin V–FITC (BD Biosciences) or Annexin V–APC (Elabscience, Wuhan, China). Both the cell cycle and apoptosis assays were assessed using a flow cytometer (BD Biosciences).

### Surviving fractions

After being treated as indicated, the cells were collected as a single cell suspension and were seeded in six-well plates at 10^3^ cells/well, with each well containing 3 mL culture medium. After incubation on the plates for 10–14 days, the colonies were washed once with PBS, fixed with 4% paraformaldehyde for at least 1 h, and were stained with crystal violet for 30 min. Colonies containing more than 50 cells were counted in 3 replicate dishes. The surviving fraction (SF) was defined as the ratio of PE (^125^I + LBP)/PE (^125^I), with PE defined as (colony number/number of seeded cells) × 100.

### Terminal deoxynucleotidyl transferase dUTP nick end labeling assay

After treatment as indicated, the cells were washed once with 1 × PBS and treated with PBS containing 0.3% Triton X-100 for 5 min. Then, the cells were fixed in 4% paraformaldehyde for at least 1 h, stained with terminal deoxynucleotidyl transferase dUTP nick end labeling (TUNEL) reagent (Beyotime Biotechnology, Nanjing, Jiangsu, China) for 1 h at 37 °C, and incubated with 4′,6-diamidino-2-phenylindole for another 15 min. Morphological changes were observed using a fluorescence microscope (Olympus, Tokyo, Japan). Three different fields were selected. Cells were counted using Image J.

### EdU staining assay

The cell proliferation was detected using Cell-Light EdU Apollo567 In Vitro Kit (RiboBio, Guangdong, China) according to the manufacturer’s instructions. After treatment, the cell medium was replaced with 50 μM EdU solution diluted with DMEM and incubated for 2 h. Hoechst 33342 was used to stain the nuclei and cell counting was done using a fluorescence microscope (Olympus, Tokyo, Japan). Cells were counted using Image J.

### Western blotting and antibodies

After treatment as indicated, the cells were washed with cold 1 × PBS, lysed with RIPA lysis buffer for 20 min on ice, and centrifuged at 12,000 r.p.m. for 10 min at 4 °C. Protein was denatured at 95 °C for 5 min. Samples (20 µg each) were analyzed by 10–12% sodium dodecyl sulfate polyacrylamide gel electrophoresis and transferred onto nitrocellulose membranes (Millipore, Burlington, MA, USA). Membranes were blocked with 5% nonfat milk in Tris-buffered saline containing 1% Tween-20 for 1 h and incubated with corresponding primary antibodies at 4 °C overnight. Then, the membranes were washed with Tris-buffered saline containing Tween-20 three times for 5 min each and incubated with corresponding secondary antibodies for 1 h at room temperature. Protein brands were detected by chemiluminescence (Millipore).

The primary antibodies anti-Bax (ab32503), anti-Bcl-2 (ab692), anti-ATF4 (ab184909), anti-CHOP (ab11419), anti-eIF2A (ab169528), anti-eIF2S (ab32157), and anti-PERK (ab79483) were purchased from Abcam (Cambridge, UK). Anti-phospho-PERK was purchased from Cell Signaling Technology (Beverly, MA, USA). The following secondary antibodies were purchased from GenScript (Piscataway, NJ, USA): goat anti-rabbit IgG (H&L), goat anti-mouse IgG (H&L), and rabbit anti-goat IgG. LBP was purchased from Changan Hainan International Pharmaceutical Co., Ltd (Haikou, Hainan, China).

### RNA isolation and qPCR

Expression levels of PERK, eIF2a (eukaryotic initiation factor 2α), ATF4 (activating transcription factor 4), and CHOP were determined using quantitative real-time PCR. Total RNA was isolated from tissues and cultured cells using TRIzol reagent (TIANGEN, Beijing, China) according to the manufacturer’s instructions. cDNA was synthesized from 1.0 μg RNA using a FastQuant RT Kit (TIANGEN). Quantitative PCR (qPCR) was performed by using BioRad C1000 Thermal Cycler CFX96 Real-Time System with SuperReal PreMix Plus (SYBR Green, TaKaRa). mRNA expression were normalized using detection of β-actin, respectively. Primers for qPCR were as follows: PERK, forward primer: 5′-ACGATGAGACAGAGTTGCGAC-3′, reverse primer: 5′-ATCCAAGGCAGCAATTCTCCC-3′; eIF2a, forward primer: 5′-CCGCTCTTGACAGTCCGAG-3′, reverse primer: 5′-GCAGTAGTCCCTTGTTAGTGACA-3′; ATF4, forward primer: 5′-ATGACCGAAATGAGCTTCCTG-3′, reverse primer: 5′-GCTGGAGAACCCATGAGGT-3′; CHOP, forward primer: 5′-GGAAACAGAGTGGTCATTCCC-3′, reverse primer: 5′-CTGCTTGAGCCGTTCATTCTC-3′; β-actin, forward primer: 5′-TCCCTGGAGAAGAGCTACCA-3′, reverse primer: 5′-AGCACTGTGTTGGCGTACAG-3′.

### Statistical analysis

Data are reported as the mean ± SD. All experiments were repeated at least twice. GraphPad Prism software version 6.0 was used for statistical analysis. One-way analysis of variance with Tukey’s multiple comparison test was utilized to analyze the subcutaneous tumor growth. An unpaired *t*-test was used when appropriate. *P *<* 0.05, **P *<* 0.01, and ***P *<* 0.001 were assumed.

## Results

### LBP increases ^125^I-induced apoptosis in HCC cells

**Fig. 1:**
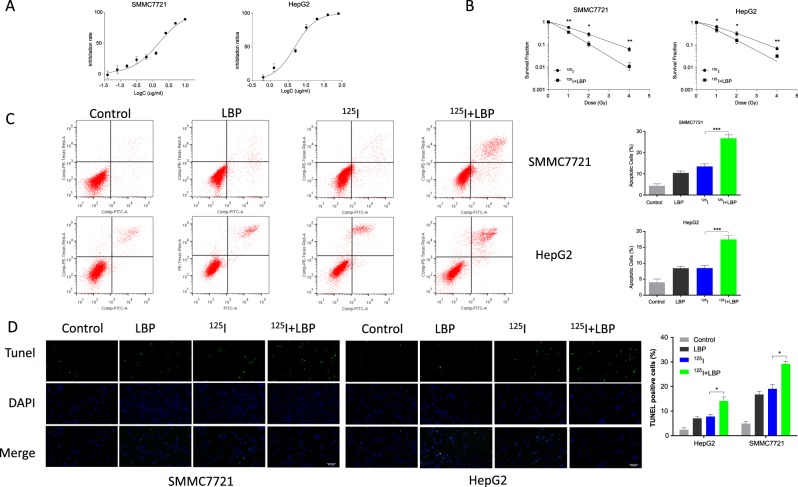
LBP increases ^125^I-induced apoptosis in HCC cells. **a** To investigate the radiosensitization effect of LBP on HCC cells to ^125^I, the concentration of LBP required for sensitization of HepG2 and SMMC7721 cell lines was determined. After treatment with LBP concentrations ranging from 0 to 80 µg/mL for 72 h, the optical density value was detected using a CCK-8 assay. The sensitization concentration was defined as 10% of IC_50_. **b** Cells were treated with ^125^I or ^125^I + LBP. After treatment, cells were plated in fresh medium for 10–14 days to assess cell survival with a colony formation assay. A single-hit multitarget model was used to construct dose-survival curves and to estimate sensitizer enhancement ratios. Based on our results, the sensitizer enhancement ratios for HepG2 and SMMC7721 cells were 1.37 and 1.63, respectively. **c**, **d** After cells were treated with ^125^I, LBP, or ^125^I + LBP, Annexin V–FITC/PI and TUNEL assays were performed to analyze cellular apoptosis. All experiments were performed in triplicate and the data are presented as the mean ± SD. The *t*-test was used for data analysis. *P < 0.05, **P < 0.01

To investigate the radiosensitization effect of LBP on HCC cells to ^125^I, the sensitization concentration of LBP was determined in HepG2 and SMMC7721 cell lines. The sensitization concentration was defined as 10% of the IC_50_. The CCK-8 assay suggested that LBP affected the viability of HepG2 and SMMC7721 cells in a dose-dependent manner. The estimated IC_50_ value of LBP at 117.2 h on HepG2 and SMMC7721 cells was found to be 5.972 µg/mL and 1.536 µg/mL, respectively (Fig. 1a).

To estimate the radiosensitizing effect of LBP, HepG2 and SMMC7721 cells were exposed to ^125^I, with and without concurrent treatment with LBP (Fig. 1b). The SF of HepG2 cells treated with ^125^I alone at 4 Gy was 0.071 ± 0.009, and that with ^125^I and LBP was 0.033 ± 0.005. The SF of SMMC7721 cells treated with ^125^I alone and with a combination of 4 Gy ^125^I and LBP was 0.061 ± 0.015 and 0.011 ± 0.005, respectively. The sensitizer enhancement ratios for HepG2 and SMMC7721 cells were 1.37 and 1.63, respectively. The radio-biological parameters of HCC cells are shown in Table 1.

**Table 1:** Radio-biological parameters of liver cancer cells

	*D*_0_ (Gy)	*N*	SF_4_	SER
HepG2				
^125^I	1.244	1.728	0.071	
^125^I+LBP	0.908	1.487	0.033	1.37
SMMC7721				
^125^I	1.307	1.361	0.061	
^125^I+LBP	0.799	1.293	0.011	1.63

The single-hit multitarget model was used to calculate the values of *D*_0,_ *N*, SF_4_, and SER. *D*_0_, mean inactivation dose; *N*, extrapolation number; SER, sensitizer enhancement ratio; SF_4_, survival fraction at 4 Gy. SER = *D*_0_ (^125^I)/*D*_0_ (^125^I + LBP).

To investigate the promotion of ^125^I-induced effects on cellular apoptosis by LBP, Annexin V–FITC/PI assay (Fig. 1c) and TUNEL assay (Fig. 1d) were performed in both HepG2 and SMMC7721 cells subjected to single or combined treatments. The Annexin V–FITC/PI assay revealed that when the HCC cells were treated with a combination of ^125^I and LBP, cellular apoptosis was significantly higher than that observed when the cells were treated with ^125^I or LBP alone (Fig. 1c). Furthermore, TUNEL assay results showed that LBP increased the ^125^I-induced cellular apoptosis in both HepG2 and SMMC7721 cells (Fig. 1d). The apoptosis was increased in the combination treatment group compared with that in the treatments with a single agent. Interestingly, the SMMC7721 cells showed more positive results than HepG2, which was consistent with the results of the radiosensitizing effect.

### LBP promotes ^125^I-induced anti-proliferative effect in HCC cells

**Fig. 2:**
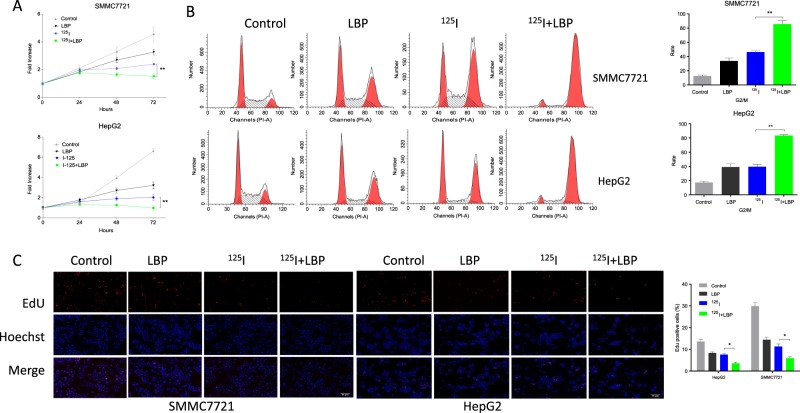
LBP promotes ^125^I-induced anti-proliferative effect in HCC cells. **a** To investigate whether the ^125^I-induced anti-proliferative effect in HCC cells was promoted by LBP, proliferation of both HepG2 and SMMC7721 cells treated with ^125^I, LBP, or a combination of the two was detected by CCK-8 assay. **b**, **c** Cell cycle and EdU assay were performed to verify the anti-proliferation effect of LBP and ^125^I on HepG2 and SMMC7721 cells. According to our results, the inhibition of cell growth was more significant in the combined treatment group than in the single treatment group. All experiments were performed in triplicate and the data are presented as the mean ± SD. The *t*-test was used for data analysis. *P < 0.05, **P < 0.01

To investigate whether the ^125^I-induced anti-proliferative effect on HCC cells was promoted by LBP, a cell proliferation assay, cell cycle assay, and EdU assay were performed for both HepG2 and SMMC7721 cells treated with ^125^I, LBP, or a combination of the two (Fig. 2). Results of the cell proliferation assay suggested that the growth of HepG2 and SMMC7721 cells was inhibited following treatment with ^125^I or LBP alone. However, the inhibition of cell growth was more significant in the combined treatment group (Fig. 2a). Furthermore, the results of cell cycle analysis showed that both ^125^I and LBP induced cell cycle arrest at the G2/M transition. In addition, the combined treatment resulted in more obvious cell cycle arrest (Fig. 2b). Besides, results of the EdU assay showed that the combination treatment clearly exhibited fewer cells with proliferative status (Fig. 2c).

### ^125^I induces upregulation of the PERK-eIF2a-ATF4-CHOP pathway to promote apoptosis

**Fig. 3:**
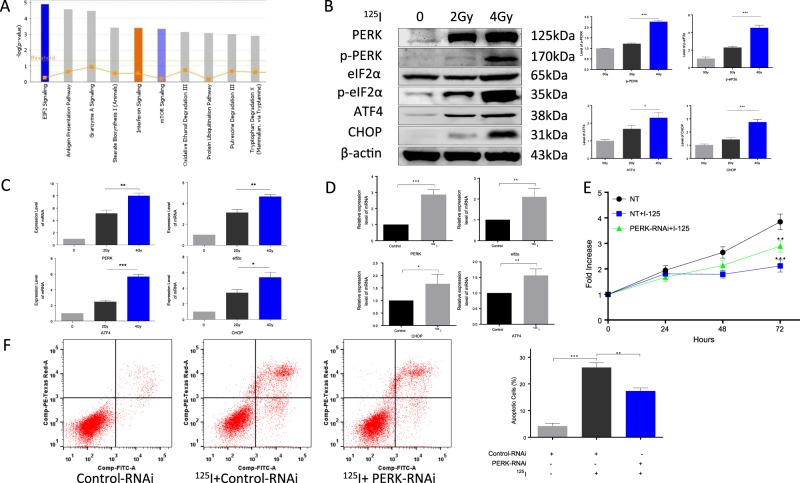
^125^I induces upregulation of PERK-eIF2a-ATF4-CHOP pathway to promote apoptosis. **a** To explore the possible apoptosis-related pathways induced by ^125^I, iTRAQ was performed in HepG2 cells. The results of the signal pathway enrichment analysis of differentially expressed proteins reveals that the eIF2 signaling pathway is significantly different between the control HepG2 cells and ^125^I-treated HepG2 cells. **b**, **c** After HepG2 cells were treated with different doses of ^125^I, the proteins and mRNA levels of the PERK-eIF2-ATF4-CHOP pathway were upregulated in a dose-dependent manner, as detected by WB (**b**) and qPCR (**c**). **d** The mRNA levels consistently increased in animal models in a dose-dependent manner, after ^125^I irradiation. Total RNA was isolated from mouse tumors and the pathway-related genes were seen to be upregulated at the mRNA level, as detected by qPCR. **e**, **f** The ^125^I-induced apoptosis and anti-proliferation effect were compromised by PERK-RNAi. After HepG2 cells transfected with Control-RNAi or PERK-RNAi were treated by ^125^I, the apoptosis and anti-proliferation effect were detected by cell proliferation assay and Annexin V–FITC/PI assay. The PERK-RNAi abrogated ^125^I-induced apoptosis and anti-proliferation effect. All the experiments were performed in triplicate and the data are presented as the mean ± SD. The *t*-test was used for data analysis. *P < 0.05, **P < 0.01

We hypothesized that ^125^I radioactive seeds induce apoptosis and inhibit proliferation of HCC cells via multiple pathways, including regulation of apoptosis-related proteins (Fig. 3). To test this hypothesis, iTRAQ was performed in control and ^125^I-treated HepG2 cells. Among the differentially expressed proteins, we selected those involved in the eIF2 signaling pathway, which was the most enriched pathway. Furthermore, we identified the PERK-eIF2α-ATF4-CHOP pathway, a well-known ER stress pathway, to be potentially involved in ^125^I-induced apoptosis (Fig. 3a).

To confirm the relationship between ^125^I and the PERK-eIF2α-ATF4-CHOP pathway, pathway-related proteins were detected after HepG2 cells were treated with ^125^I. The results of western blotting and qPCR demonstrated that the expression levels of the selected protein and mRNA were significantly increased in a dose-dependent manner (Fig. 3b, c). Similar results were obtained in the mouse tumor model. The expression of the pathway-related genes was also upregulated at the mRNA level, as detected by qPCR (Fig. 3d). Furthermore, to verify whether ^125^I induces apoptosis through the PERK-eIF2α-ATF4-CHOP pathway, PERK-RNAi and Control-RNAi were transfected into HepG2 cells. After treatment with ^125^I, a cell proliferation assay was performed to evaluate the effect of ^125^I on anti-proliferation (Fig. 3e). According to our results, the PERK-RNAi abrogated the ^125^I-induced anti-proliferation effect. Apoptosis was detected by the Annexin V–FITC/PI assay. Compared with the cells transfected with Control-RNAi, the apoptosis rate was reduced in PERK-RNAi-transfected cells. Taken together, these data indicate that ^125^I induces the upregulation of the ER stress pathway to promote apoptosis (Fig. 3f).

### LBP increases the ^125^I-induced upregulation of the PERK-eIF2α-ATF4-CHOP pathway to promote ^125^I-induced apoptosis

**Fig. 4:**
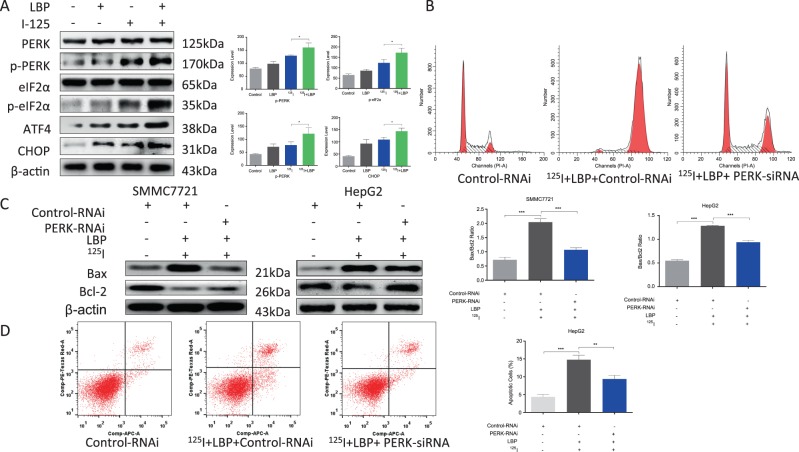
LBP increases ^125^I-induced upregulation of PERK-eIF2α-ATF4-CHOP pathway to promote ^125^I-induced apoptosis. **a** LBP boosts the ^125^I-induced upregulation of the PERK-eIF2α-ATF4-CHOP pathway. To further confirm the role of LBP as a sensitizer in ^125^I-mediated apoptosis and anti-proliferative effect in HCC cells, the PERK-eIF2α-ATF4-CHOP pathway-related proteins were detected by WB after the treatment of SMMC7721 cells with ^125^I or a combination of ^125^I and LBP. **b**–**d** The combination of ^125^I and LBP induced apoptosis and anti-proliferative effect on HCC cells that could be compromised by PERK-RNAi. After SMMC7721 and HepG2 cells transfected with Control-RNAi or PERK-RNAi were treated with ^125^I and LBP, the cell cycle (**b**), Bax/Bcl-2 ratio (**c**), and Annexin V–APC/PI assay (**d**) were performed to verify the rescue effect on apoptosis and anti-proliferation. All the experiments were performed in triplicate and the data are presented as the mean ± SD. The *t*-test was used for data analysis. *P < 0.05, **P < 0.01

To further confirm the sensitizer role of LBP in ^125^I-mediated apoptosis and anti-proliferative effect in HCC cells, the PERK-eIF2α-ATF4-CHOP pathway-related proteins were detected after SMMC7721 cells were treated with ^125^I or a combination of ^125^I and LBP (Fig. 4a). The results showed that the expression levels of the selected proteins were significantly increased in the combination treatment group compared with their levels in the group that was treated with ^125^I alone.

To further verify the role of the PERK-eIF2α-ATF4-CHOP pathway in the ^125^I and LBP coinduced apoptosis and anti-proliferation of HCC cells, cell cycle (B), Bax/Bcl-2 ratio (C), and Annexin V–APC/PI assays (D) were performed. After SMMC7721 and HepG2 cells were transfected with Control-RNAi or PERK-RNAi, cells were treated with ^125^I and LBP. According to the results, the apoptosis and anti-proliferation induced by the combination of ^125^I and LBP in HCC cells could be compromised by PERK-RNAi. Compared with the cells transfected with Control-RNAi, the G2/M rate, Bax/Bcl-2 ratio, and apoptosis rate were decreased in cells transfected with PERK-RNAi. Taken together, these results suggested that LBP promotes ^125^I-induced apoptosis via increasing the ^125^I-induced upregulation of the PERK-eIF2α-ATF4-CHOP pathway.

### PERK downregulation compromises the inhibition of HCC growth induced by ^125^I and LBP in nude mouse SMMC7721 xenograft tumors

**Fig. 5:**
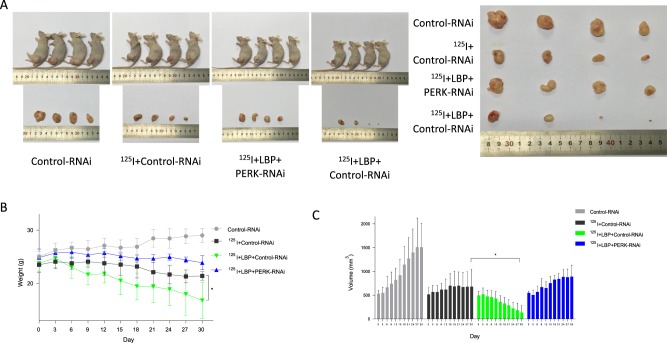
PERK downregulation compromises the inhibition of HC growth induced by ^125^I and LBP in SMMC7721 xenograft tumors in nude mice. To further verify the effect of the PERK-eIF2α-ATF4-CHOP pathway on ^125^I-mediated apoptosis and anti-proliferation of HCC cells, SMMC7721 cells transfected with PERK-RNAi or Control-RNAi xenograft tumors were treated with ^125^I and LBP. When the volume of the tumors reached 500 mm^3^, the mice were randomly divided to three groups with four mice in each group. After 30 days of treatment, the mice were killed and the tumors were exfoliated. The tumor weight (**b**) and diameters (**c**) were measured every other day for 30 days. One-way ANOVA with Tukey’s multiple comparison test was utilized to analyze the subcutaneous tumor growth. All the experiments were performed in triplicate and the data are presented as the mean ± SD. The *t*-test was used for data analysis. *P < 0.05, **P < 0.01

To verify the role of the PERK-eIF2α-ATF4-CHOP pathway in ^125^I-mediated apoptosis and anti-proliferation, SMMC7721 xenograft tumors were treated with ^125^I, LBP, and RNAi (PERK-RNAi and Control-RNAi). The results showed that, compared with the ^125^I treatment alone, the combination treatment inhibited the tumor growth more significantly (Fig. 5). As shown in the Fig. 5a, the combination of ^125^I and LBP inhibited tumor growth more cooperatively than ^125^I treatment alone. However, when the cells were transfected with PERK-RNAi, inhibition of tumor growth was reduced compared with that in the cells transfected with Control-RNAi. Consistent with the diminished growth curves, tumor weights at the time of killing were also significantly reduced in mice treated with the combined treatment compared with mice treated with ^125^I alone (Fig. 5b). Moreover, the tumor volume was more obviously decreased by the combined treatment than by treatment with ^125^I alone (Fig. 5c). In general, these data indicated that LBP promotes ^125^I-induced anti-proliferation in liver cancer cells. Overall, these results indicate that LBP increases the ^125^I-induced upregulation of the PERK-eIF2α-ATF4-CHOP pathway to promote apoptosis and the inhibition of proliferation.

## Discussion

^125^I seed implantation has recently become a standard treatment for the management of HCC^12,16–18^. The risk of radiological complications is correlated with the dose, treatment volume, and dose rate^19^. A cardinal principle of ^125^I seed implantation is to use the minimal dose, also known as the biologically effective dose, at which tumor cells are killed but neighboring normal cells are spared. Decreasing the total dose is a feasible method for preventing radiological complications. In a previous study, we showed that ^125^I induces apoptosis and inhibits proliferation in pancreatic cancer cells^20^. However, the mechanism by which ^125^I induces apoptosis and inhibits proliferation in HCC is still unknown. Based on the results of iTRAQ, eIF2 signaling pathway was found to be the most enriched pathway. We identified the PERK-eIF2a-ATF4-CHOP pathway to potentially participate in ^125^I-induced apoptosis. Here we investigated whether the radiosensitivity of HCC to ^125^I could be promoted by LBP and the possible links between ^125^I, LBP, and PERK-eIF2α-ATF4-CHOP pathway.

LBP has been approved for the treatment of several cancers, including liver, stomach, lung, and nasopharyngeal cancers, and the combination of ^125^I seeds and LBP results in improved prognosis in the treatment of hepatic cancer^21–23^. A retrospective study showed that TACE with gelatin sponge microparticles mixed with LBP is a safe and effective method for stage B HCC patients^12^. Futhermore, it was reported that the combination of LBP-TACE and ^125^I radioactive seeds results in a better overall survival than LBP-TACE therapy alone; thus, a comprehensive therapy is recommended for these patients^13^. The present study indicates that LBP causes significant apoptosis and inhibition of proliferation in HCC cells, similar to that observed in ^125^I seed RT alone, whereas a combination of the two treatments results in an even greater effect in vitro and in vivo. These results indicate that LBP promotes ^125^I-induced apoptosis and inhibition of proliferation in HCC.

A possible pathway involved in the ^125^I-mediated apoptosis and inhibition of proliferation in HCC cells could be the PERK-eIF2α-ATF4-CHOP pathway. The activated PERK elicits UPR-related pro-apoptotic signals, markedly elevating the levels of phosphorylated eIF2α. This results in the activation of a pro-adaptive signaling pathway via the inhibition of global protein synthesis and selective translation of ATF4, a transcription factor that regulates many genes related to the recovery of cells and their adaption to stress^24^. However, ATF4 was found to be related to 5-fluorouracil resistance in colorectal cancer^25^. Interestingly, the ATF4 levels were upregulated after treatment with gemcitabine in pancreatic cancer cells and could be regarded as a mechanism for induction of apoptosis^26^. Furthermore, ATF4 increases the expression levels of transcription factor CHOP. Overexpression of CHOP downregulates the expression of Bcl-2, upregulates Bax, and evokes apoptosis^7^. In addition, CHOP plays an important role in another pro-apoptotic mechanism, because it directly activates the growth arrest and DNA damage-inducible protein, GADD34, which sufficiently promotes the dephosphorylation of eIF2α and reboots protein translation in stressed cells^27^. Our results showed that ^125^I upregulated the PERK-eIF2α-ATF4-CHOP pathway in a dose-dependent manner. Besides, the apoptosis induced by ^125^I was reduced when PERK was downregulated by PERK-siRNA. More importantly, not only did LBP enhance the upregulation of the PERK pathway induced by ^125^I, the inhibition of tumor growth in mouse tumor model treated with combined treatment was compromised when PERK-siRNA was transfected into HCC cells, which might explain the mechanism by which LBP promotes the radiosensitivity of HCC cells to ^125^I.

Our results also show that LBP significantly increased the ^125^I-induced cell cycle arrest in HepG2 cells. The ratio of cells in the G2/M stage in the combination treatment group was markedly higher than that in the ^125^I seed radiation group. Univariate general linear model analysis showed that the arrest of HCC cells was greater in the combination treatment group than in the groups treated with either of the agents alone, indicating that LBP might have a synergistic effect with ^125^I seeds. Dai et al.^8^ reported that cell cycle arrest might be associated with the downregulation of CDK1, phosphorylated CDK1 (p-CDK1), cyclin B, CDC25C, p-CDK4, Rb, p-Rb, and E2F, and the upregulation of p27, p21, and p53^8^. Besides, cells at G2/M are more sensitive to radiation, which might explain the ability of LBP to promote radiosensitivity of HCC cells to ^125^I seeds.

The data in this study indicate that LBP promotes the radiosensitivity of HCC cells to radioactive ^125^I seeds. In addition, when comparing the radiosensitivity of HepG2 and SMMC7721 cells with ^125^I seeds, it is interesting to find that the latter are more sensitive to ^125^I seeds. The sensitization enhancement ratio for HepG2 and SMMC7721 was 1.37 and 1.63, respectively, indicating that a lower dose could be used to kill hepatic cancer cells when ^125^I seed radiation is combined with concurrent LBP chemotherapy. We believe that the different effect between the two cell lines could be due to the different expression levels of ZHX2. ZHX2 has been reported to function as a tumor suppressor in the development of HCC and the reduced ZHX2 level also leads to a lower response to chemotherapeutic drugs^28–30^. Thus, further study is needed to investigate the link between ^125^I seeds, radiosensitivity, and ZXH2 ZHX2.

## Conclusion

In summary, the data in this study reveal a relationship between ^125^I, LBP, and the PERK-eIF2α-ATF4-CHOP pathway. LBP promotes ^125^I-induced apoptosis and inhibition of proliferation of HCC by upregulating the PERK-eIF2α-ATF4-CHOP pathway. Furthermore, our data lays a solid foundation for better clinical application of this combined therapy.

## Data Availability

All authors declare that all data and materials described in the manuscript will be freely available to any scientist wishing to use them for non-commercial purposes.
